# Cultural Adaption, Translation, Preliminary Reliability and Validity of Key Psychological and Behavioural Measures for 18 to 25 Year-Olds Living with HIV in Uganda: A Multi-Stage Approach

**DOI:** 10.1007/s10461-023-04193-y

**Published:** 2023-10-04

**Authors:** Michael Evangeli, Caroline Foster, Victor Musiime, Sarah Fidler, Janet Seeley, Graham Frize, Annette Uwizera, Joseph Price

**Affiliations:** 1 Department of Psychology, Royal Holloway University of London, Egham, Surrey TW20 0EX UK; 2 Department of Paediatric Infectious Diseases, Imperial College Healthcare NHS Trust, London, UK; 3 Department of Paediatrics and Child Health, Makerere University, Kampala, Uganda; 4 Research Department, Joint Clinical Research Centre, Kampala, Uganda; 5 Department of Infectious Disease, Imperial College London, Imperial College NIHR BRC, London, UK; 6 Department of Global Health and Development, London School of Hygiene and Tropical Medicine, London, UK; 7 Central and North West London NHS Foundation Trust, London, UK

**Keywords:** Measures, HIV, Young adults, Uganda, Validity

## Abstract

HIV remains a significant public health issue among young adults living in Uganda. There is a need for reliable and valid measures of key psychological and behavioural constructs that are related to important outcomes for this population. We translated, adapted and tested the psychometric properties of questionnaires measuring HIV stigma, HIV disclosure cognitions and affect, antiretroviral therapy (ART) adherence, social support, personal values, and hope, using a multi-step process. This included: translation, back-translation, expert review, cognitive interviewing, readability and assessments of internal consistency with 93 young adults (18–25 years) living with perinatally acquired HIV in Uganda. Preliminary criterion validity was assessed by examining relationships between the adapted measures and wellbeing, HIV disclosure behaviour, HIV disclosure intention and viral load suppression. The measures all showed acceptable reliability and every questionnaire apart from the Agentic and Communal Value Scale was easy to read. Those scales measuring HIV disclosure affect and cognitions, social support, HIV stigma and hope showed relationships with other constructs suggestive of validity. There is preliminary evidence to support the use of these measures in research and clinical contexts for young adults living with perinatally acquired HIV in Uganda.

**Supplementary Information:**

The online version contains supplementary material available at 10.1007/s10461-023-04193-y.

## Introduction

Globally, young people aged 15–24 years account for up to 27% of new HIV infections [1]. Approximately 78% of this population live in sub-Saharan Africa [2]. Uganda has an estimated prevalence rate of 5.4% in those aged 15–49 years, and an incidence rate per 1000 people of 0.95% [1]. Young people aged 15 to 24 years represent 21% of the country’s population and make up 12% of those living with HIV [3, 4]. Many of these young people live with perinatally acquired HIV (PAH) and are the long-term survivors of the perinatal epidemic. Young people with PAH face a number of unique challenges to their psychological well-being [5]. There have been calls in the last decade to develop both behavioural interventions and culturally appropriate measures to address and assess these challenges globally, the latter because existing versions are typically only standardised for English-speaking, high income, populations [6, 7].

Antiretroviral therapy (ART) adherence is the most important factor for achieving viral suppression if medication is available [8]. In 2021 treatment coverage was approximately 83% in Uganda [9]. Young people with PAH are now transitioning from paediatric to adult care in Uganda [10]. However, many adolescents have had difficulties maintaining good ART adherence; difficulties which can continue into early adulthood [11–13]. Greater psychological distress, poorer mental health, poorer quality of life, depression, and anxiety are all associated with lower ART adherence levels [14–19]. Accurate self-report measurement of ART adherence (alongside viral load measurement) is important to identify at-risk individuals and maintain ART adherence. Self-report ART adherence measures have been used in research studies in Uganda [20], although it is unclear if commonly used questionnaires (e.g., the CASE Adherence Index) [21] are linguistically and culturally valid. A reliable, valid and culturally appropriate measure would be a valuable tool for HIV researchers and for healthcare professionals working with young people living with HIV in Uganda.

Onward HIV disclosure (or self-disclosure) refers to the sharing of one’s HIV status with others. Disclosure rates among young people with PAH are lower than their peers with behaviourally acquired HIV [22] and those living with PAH have the additional complexity of potentially disclosing the status of other family members. Many individuals report anxiety about HIV disclosure [23], with perceived risks of HIV-status sharing, alongside potential behavioural and psychological benefits. Regarding the latter, onward HIV disclosure has been associated with both higher levels of ART adherence, in a number of studies [24–27], and increased social support [20]. The Adolescent HIV Disclosure Cognition and Affect Scale (AHDCAS) was recently developed to measure disclosure attitudes, beliefs, and self-efficacy. The scale showed good levels of reliability and preliminary criterion and construct validity in a sample of British adolescents (aged 12–16) living with HIV. Participants who had shared their status showed more positive disclosure attitudes and higher levels of disclosure self-efficacy (confidence in being able to share their HIV status with others). In addition, those with higher levels of disclosure intention showed more positive disclosure attitudes and feelings [28]. However, there is no culturally appropriate scale measuring disclosure attitudes and beliefs for young people in Uganda. Translating and adapting the AHDCAS is a logical next step in understanding the disclosure decision-making process in this particular cultural context.

High levels of HIV stigma have been associated with reduced quality of life and lower psychological well-being in sub-Saharan Africa [29, 30]. This includes higher levels of depressive symptoms [31, 32] and suicidal thoughts [33, 34]. HIV stigma is also a major barrier to ART adherence and onward disclosure [35] preventing the potential positive outcomes associated with these behaviours. Young people in sub-Saharan Africa living with HIV are vulnerable to the adverse effects of HIV stigma, particularly internalised stigma [32]. A short version of the HIV Stigma Scale [36] measuring HIV-related negative self-image was recently developed in a Swedish sample of young people with HIV [37], but a valid and reliable measure of internalised HIV stigma is not available for Uganda. The development of a short, standardised measure would be valuable for understanding the important role of internalised HIV stigma in this cultural context.

Social Support is an important protective factor against poor psychological wellbeing. Studies of young people with HIV in Uganda have shown positive associations between social support and good psychological and behavioural outcomes. For example, greater levels of social support have been associated with less psychological distress in adolescents living with HIV [38], and less depression and anxiety in women living with HIV [39]. Social support from family/caregivers has been associated with higher levels of ART adherence in adolescents with HIV [40]. Lower levels of social support (using a measure translated into Runyankole, a language spoken mainly in western Uganda) have also been associated with higher levels of internalised and enacted HIV stigma [41]. A range of social support measures have been used in the above studies but there is not a brief social support scale that has been developed for young adults, in Luganda, the commonly spoken language in central Uganda, where Kampala is located (and where the study participants were drawn from). Luganda is also the most widely indigenous language in Uganda. Producing a valid and reliable social support measure is therefore vital to fill this gap.

Personal values are defined as “desirable trans-situational goals, varying in importance, that serve as guiding principles in the life of a person” (42). Higher ratings of health values were associated with lower ratings of depression and anxiety in asthma (43). Higher ratings of living consistently with one’s values were associated with lower ratings of anxiety and depression in cancer patients [44]. Personal values can be grouped into two broad types. Agentic values are *‘self-focused’* and characterised by independence, self-direction, and personal achievement. Communion values are *‘other-focused’* and characterised by social belonging, trustworthiness, and equality. The agentic and communal value scale (ACV) is a reliable and valid measure commonly used in research [45]. Higher ratings of agentic and communion value behaviours have been associated with higher ratings of psychological well-being in young adults [46]. Only one study has used the ACV scale to investigate the relationship between personal values and HIV in British young adults with PAH with a mean age of 23.8 years [47]. The results showed that higher scores of agentic values were associated with lower disclosure *intention*, whilst greater communion values were associated with higher disclosure *rate*. Understanding the relationship between HIV, well-being, and personal values requires a valid, reliable, and culturally appropriate version of the ACV to explore these associations in other contexts.

Hope is often recognised as a multidimensional construct reflecting an individual’s goals, the pathways to those goals, and the motivation to achieve those goals [48]. Higher levels of hope have been associated with higher quality of life in children with cancer [49]. It may be that hope is relevant for HIV illness coping behaviour such as medication adherence. There is, however, no standardised and reliable measure of state hope for young adults living with HIV in Uganda, and studies have suggested that existing scales are not valid for use in sub-Saharan Africa [50].

As illustrated, there are relationships between the six domains discussed (ART adherence, HIV disclosure, HIV Stigma, social support, personal values, and hope) and psychological well-being in young people with PAH. However, there are a lack of reliable and valid measures for this population in Uganda. Measures designed for one language or culture may fail to accurately capture the same psychological construct in another language or culture. Simple translation of pre-validated measures from English-speaking, high income, contexts neglect conceptual or operational differences between cultures [51–54]. Based on previous literature, the following measures we adapted were the CASE Adherence Index [55], the Adolescent HIV Disclosure Cognition and Affect Scale [56], the negative self-image subscale from the short form HIV Stigma Scale [37], the Social Support Questionnaire short form – SSQ6 [57], the Agentic and Communal Value Scale (ACV) [45], and the State Hope Scale [58].

The aim of this paper is to report the process of cultural adaptation and development of measures for key psychological domains relevant to psychological well-being, in the context of piloting a disclosure intervention for PAH young people living in Uganda [59]. Cognitive interviewing established conceptual understanding and cultural appropriateness, whilst quantitative analysis established reliability (internal consistency), preliminary construct validity (the scale accurately measuring the psychological construct it was intended for), and criterion validity (the scale correlating with a conceptually related external measure, for example, positive disclosure attitudes correlating with actual HIV disclosure).

## Methods

### Participants

Three samples from a two-phase HIV disclosure intervention study [59] were used in the development and adaptation of measures. The first sample, drawn from phase one (intervention development phase) of the study, consisted of 10 young people (3 male, 7 female) from Uganda attending the Joint Clinical Research Centre (JCRC), Lubowa, on the outskirts of Kampala, the capital city of Uganda. JCRC is a centre of excellence for health care and research providing services to over 10,000 people living with HIV (https://jcrc.org.ug/). In this study, participants were invited to participate based on the following eligibility criteria: aged 18 to 25 years old (median age 22, range 21–25), living with PAH, receiving care at the study site, knowledge of their own HIV-positive status and able to give informed consent. Exclusion criteria included current serious mental health issues, moderate to severe learning difficulties, serious physical health problems with a life expectancy < 12 months, or an inability to understand and communicate in either English or Luganda. Participants were compensated 50,000 Ugandan shilling for their time and travel expenses incurred.

A second sample of local experts were recruited to assist in the translation and adaptation of measures. Two paediatricians and one psychologist from JCRC were recruited to form an expert review panel based on the following criteria: able to understand and communicate in *both* English and Luganda, conceptual and cultural knowledge relevant to the target population, and a professional background in HIV and working with young people.

The third sample, drawn from phase two of the HIV disclosure intervention study, consisted of 93 young people (56 female, 37 male) from Uganda. 153 young people were approached. The same inclusion criteria as sample one were used. The same exclusion criteria were used with one additional criterion: participation in phase one of the HIV Disclosure intervention study. All participants were compensated for their time and travel expenses incurred. Sample demographics, disclosure and clinical characteristics for this sample are presented in Table 1. All participants were born in Uganda.

**Table 1:** Sample demographic, disclosure and clinical characteristics of sample 3

	Frequency (%)
**Gender**	
Male	37 (39.8)
Female	56 (60.2)
**Age (in years)**	
Mean (sd)	22.09 (2.05)
**Tribe**	
Baganda	63 (67.7)
Banyankore	6 (6.5)
Basoga	4 (4.3)
Bakiga	4 (4.3)
Bagisu	3 (3.2)
Iteso	3 (3.2)
Other	10 (10.8)
**Lifetime HIV Disclosure (number disclosed to)**	
0	11 (11.8)
1–3	36 (38.7)
4–9	17 (18.3)
10+	25 (26.9)
Missing	4 (4.3)
**HIV Disclosure in last 6 months (number disclosed to)**	
0	80 (86.0)
1	5 (5.4)
2+	8(8.7)
**On ART**	
Yes	93 (100.0)
**Viral Load (copies/mL)**	
<200	82 (88.2)
>200	7 (7.5)
Missing	4 (4.3)

### Ethics

Ethical Approval was granted by a UK NHS Research Ethics Committee, the Royal Holloway, University of London College Ethics Committee, the Joint Clinical Research Centre Research Ethics Committee and Uganda National Council of Science and Technology. Informed written consent was obtained for all participants.

### Procedure

The following 6-step procedure was used:

1. *Translating existing questionnaires/items and response options*. The aim of this first stage was for conceptual translation (not word for word translation, but culturally accurate translation) by a translator to produce simple, clear and concise items and response options appropriate for the target group (18- to 25-year-olds living with PAH in Uganda).
2. *Back translation*. Items were back translated by a different translator.
3. *Review*. The original and back-translated items were compared by the research team. Where differences existed, these were forwarded to the expert panel to assess the cultural and linguistic appropriateness of the translations, comparing original measures, the Luganda translation and the English back-translated measures. Expert review participants answered the following closed question for each item: “*Are you happy with this translation?”* with a simple *Yes-No* response. If *yes*, then the translations were deemed appropriate. If *no*, the expert review participants were asked to consider which of the following options best captured the issue with the back-translated item: [1] no equivalent local concept in Luganda, [2] a meaning narrower than the original, [3] a meaning expanded beyond the original, [4] a cultural applicability problem, [5] the translation is not clear, resulting in an inaccurate backtranslation or [6] other. Finally, expert reviewers were invited to suggest revised Luganda translations of the original English.
4. *Cognitive interviewing* [60]. The ten young people comprising sample 1 were split into two equal groups. Each group were interviewed about three of the six measures. All interviews were audio-recorded. The interviewer used a concurrent verbal probing approach to assess participants’ thought processes in the following six domains after the questions were asked and answered:
  - difficulty in understanding the question (*how hard was it to answer?*)
  - understanding through paraphrasing (*can you repeat the question in your own words?*)
  - comprehension of questions and response options in English and Luganda (*what do you think this question is about?*)
  - confidence in one’s answer (*how sure are you of your answer?*)
  - recall of relevant factual information from memory (*how do you remember the answer to this question?*)
  - perceived validity (*how likely is it that people would answer this question truthfully?).*

Questions were asked and answered by participants.

5. *Amendment of measures*. Based on feedback from the cognitive interviewing stage, amendments were discussed and agreed on by the research team. The measures were formatted with English and Luganda wordings next to each other.
6. *Administration of measures*. The measures (as well as other measures used to assess criterion validity) were administered in a face-to-face group format at the beginning of the disclosure intervention study (baseline) to sample three participants. For all of the target measures, participants read each question and recorded their responses individually. Data collected from this stage was used to determine the reliability and validity of all adapted measures.

### Target Measures

#### ART CASE Adherence

The CASE Adherence Index (55) was used to measure self-reported adherence behaviour. The measure contains three items that measure difficulty in taking ART medication on time, scored from (4) *never* to (1) *all the time*, frequency of missed doses per week scored from (1) *every day* to (6) *never*, and time since most recent missed dose scored from (1) *within the past week* to (6) *never*. The minimum score is 3 and the maximum is 16. Scores from each item are summed, with a total score greater than 10 indicating good adherence, whilst a score of 10 or less indicates poor adherence. The measure has been used previously in Uganda, but no adaptation process was used to establish reliability and validity in this cultural setting (20). Previous work in adults living with HIV has demonstrated the measure’s reliability, criterion validity and sensitivity to change using three-day adherence self-report data and comparing it to changes in HIV virologic outcomes and CD4 counts across four time points [55].

#### Adolescent HIV Disclosure Cognition and Affect

The Adolescent HIV Disclosure Cognition and Affect Scale [56] was used. This 18-item scale measures negative disclosure attitudes and feelings, positive disclosure attitudes and feelings and disclosure self-efficacy, and has demonstrated good reliability (α = 0.79) and validity in a sample of UK adolescents living with PAH. An example item is *“I am confident that I can deal with how others respond if I share my HIV status with them”*. Responses are made on a five-point Likert scale from (1) *strongly disagree* to (5) *strongly agree*. The minimum score is 18 and the maximum is 90, with higher scores indicating more positive sharing attitudes, feelings about sharing, and sharing self-efficacy. An additional HIV disclosure intention item, “*I intend to tell someone new about my HIV status in the next 6 months*”, using the same response options, forms the complete measure. Higher scores indicate greater intention to disclose.

#### HIV Stigma

The negative self-image subscale from the short form HIV Stigma Scale (37) was used. This scale contains three items that measure self-stigma and has demonstrated good reliability (α = 0.80) in a Swedish sample of adults living with HIV. An example item is *“I feel guilty because I have HIV”*. Responses range from [1] *strongly disagree* to [4] *strongly agree*. The minimum score was 3 and the maximum was 12, with higher scores indicating higher levels of self-stigma.

#### Social Support

The Social Support Questionnaire short form – SSQ6 (57) was used. This measure contains six items asking participants to first consider who in their life provides social support, and then rate how satisfied they are with the overall support these people provide. An example item is *“Who can you really count on to be dependable when you need help?”.* Responses are made on a scale ranging from [1] *very dissatisfied to* [6] *very satisfied*. The minimum score on the satisfaction subscale is 6, whilst the maximum score is 36, with higher scores indicating higher social support satisfaction. The measure has demonstrated good reliability (α = 0.89) in young people living with HIV from the United States [61]. A longer form of this measure has been used with young people living with HIV in Uganda [62], but a valid and reliable short form will be a valuable alternative for future research.

#### Values

The Agentic and Communal Value Scale (ACV) (45) was used. This 24-item measure lists 12 agency items (e.g., wealth, achievement) and 12 communion items (e.g., trust, compassion). Participants rate the importance of each item as *“a guiding principle in my life”* from [1] *not important to me* to [9] *highly important to me*. Scores range from 12 to 108 for both subscales with higher scores indicating greater importance of agency and communion values. The measure has been used with British young adults [46, 63], including those with PAH [64], and there is evidence of good reliability for both subscales (agentic α = 0.71; communal α = 0.66) [47].

#### Hope

The State Hope Scale [58] was used. The questionnaire contains six items measuring current levels of hope to proximal events in one’s life on a scale from (1) *definitely false* to (8) *definitely true*. An example item is *“There are lots of ways around any problem that I am facing now”*. The scale has demonstrated good reliability in young adults from the US (α = 0.93). The minimum score is 6 whilst the maximum score is 48, with higher scores indicating greater levels of agency and goal achievement. The trait version of this hope scale has been previously adapted and translated into Luganda [65].

### Additional Measures to Assess Criterion Validity

#### Wellbeing

The 6-item psychological domain from the World Health Organisation Quality of Life brief questionnaire (WHOQOL BREF) was used [66]. This measure has been translated into Luganda with good evidence of reliability and validity [67]. It includes questions on bodily image and appearance, negative feelings, positive feelings, self-esteem, spirituality/religion/personal beliefs, and thinking/concentration, and was answered on a 5-point scale (e.g. from 1-*not at all* to 5-*completely*). Alpha in this sample was 0.76.

#### Disclosure Behaviour

Self-reported HIV sharing events were assessed through recording:

- The frequency of new disclosures in the last six months to partners, friends and family (first hand or second hand with consent). Participants were asked to generate a list of people (e.g., partners, family, friends) in their social network. For each identified person, they were asked, “*Do they know if you are HIV positive*?”. If the answer was yes, they were asked, “*How long have they known?*”. If the answer was less than 6 months, they were asked, “*Did you tell them yourself?*”.
- The frequency of lifetime disclosure (*How many people have you told about your HIV status?*) with the following response options – 0, 1, 2, 3, 4–9, 10+. Data was also grouped into two categories – 0 to 3 inclusive, and 4+.

#### Viral Load

Most recent viral load was collected from participants’ clinical records with a VL < 200 copies/ml classed as undetectable/suppressed with no risk of onward transmission to partners [68].

### Data Analysis

Data were analysed using SPSS 25. Cronbach’s alpha (internal consistency) was calculated for both the total scales and subscales of each measure adapted for the study. Published guidelines were used to interpret alpha levels (69). Preliminary criterion validity was assessed by examining the relationships between the target measures and other variables using independent t tests and Pearson’s correlations with bootstrapped confidence intervals where parametric assumptions were not met. Two-tailed tests were used. Effect sizes for mean differences between groups were calculated [70] and interpreted in relation to published guidelines [71].

## Results

After translation, back-translation and review by the research team, eight items were forwarded to the expert review panel as being problematic: one item from the HIV stigma questionnaire, “*People’s attitudes about HIV make me feel worse about myself”* was back translated to, “*I am fed up with how people treat those living with HIV”;* six items from the Personal Values questionnaire, for example, the original English version of *“INFLUENCE (having impact, influencing people and events)”* was originally back translated to *“To encourage (To be of use, to encourage others)”;* one item from the Hope questionnaire, *“Right now, I see myself as being pretty successful”* was back translated to *“At the moment, I see that I have many opportunities”.* Revisions to items were offered by the expert review panel to remedy the above problems.

The cognitive interviewing stage showed that the Social Support, Values and State Hope scales required additional item modifications to address confusing or contextually irrelevant information. Changes were made to either the English or Luganda translation as necessary. The following amendments were made with interviewers’ representative quotes presented in Table 2 for all constructs:

**Table 2:** Quotations illustrating need to adapt items

Construct	Item	Representative Quote
**Social Support**	3. Who accepts you totally, including both your worst and best points?	*“Worst moments when disclosing her status and best moment when she felt free after telling someone”.* Does not refer to personal characteristics.
	6. Who can you count on to console you when you are very upset	*“(participant) understood consoling as counselling”.*
**Values**	8. ACHIEVEMENT (reaching lofty goals)	*“Have set many goals and wish to reach them although I have not reached all”.* Does not convey importance of achieving ambitious goals.
**Hope**	2. At the present time, I am energetically pursuing my goals	*“The word energetically was not understood by this young person…”.*
	3. There are lots of ways around any problem that I am facing now	*“Has many problems and hopes to be out of them soon”.* Does not convey that there are solutions to current problems
	4. Right now, I see myself as being pretty successful	*“See my future as bright”.* Does not convey current focus and positive self-appraisal.

1. *Social Support Scale*. Two items [3, 6] needed modifying on this scale to improve clarity. Item 3 changed from “*Who accepts you totally, including both your worst and best points?*” to “*Who accepts you totally, including both your worst and best qualities*?”. Almost all participants misunderstood the word `points’ to mean particular points in time, when the intended meaning was characteristics, qualities, or attributes. Item 6 changed from *“Who can you count on to console you when you are very upset”* to *“Who can you count on to comfort you when you are very upset”.* The majority of participants understood the word `console’ to mean counselling, when it really means to comfort. There was no change to the Luganda version for either item.
2. *Values*. One item needed modifying on this scale to improve comprehension. Item 8 changed from *“ACHIEVEMENT (reaching lofty goals)”* to “ACHIEVEMENT (reaching high goals)” in the English version with no change to the Luganda version. The majority of participants understood the word `lofty’ to mean having many goals or working hard, when its intended meaning was about having ambitious or important goals.
3. *State Hope Scale*. Three items (2, 3 and 4) needed to be modified to improve clarity and comprehension. Item 2 changed from “*At the present time, I am energetically pursuing my goals*” to “*At the present time, I am pursuing my goals with energy*” in the English version, with no change to Luganda. In many instances, the participants understood the word energetically to mean ‘high energy’, instead of `with enthusiasm’. Item 3 changed from *“There are lots of way around any problem that I am facing now”* to “*There are lots of solutions to any problems I am facing now*” in the English version. Item 4, which states “Right now, I see myself and being pretty successful” needed the word `now’ underlined, in the English version, as participants misunderstood this as referring to future prospects. There was no need for change in the Luganda version.

The Flesch reading ease score [72] for the English version of the SSQ6 was 80.9 (easy to read), 81.4 for the Adolescent HIV Disclosure Cognitions and Affect Scale (easy to read), 92.3 for the *CASE* Adherence Index Scale (very easy to read), 78.2 for the negative self-image subscale of the HIV Stigma Scale (very easy to read), 81 for the State Hope Scale (easy to read) and 43.4 for the ACV (difficult to read). Final versions of the target measures are included as Appendices.

Scale and subscale means, and standard deviations are presented in Table 3.

**Table 3:** Descriptive statistics for measures

Measure	Total or subscale name (possible range of scores, participants)	Mean	SD
**CASE Adherence Index**	Total ART Case Adherence (3–16, n = 90)	12.00	2.98
**The Adolescent HIV Disclosure Cognition and Affect Scale**	Total Scale (18–90, n = 67)	58.54	10.81
	*Subscale 1: negative disclosure attitudes and feelings* (8–40, n = 75)	22.57	6.26
	*Subscale 2: disclosure self-efficacy* (6–30, n = 87)	21.26	6.38
	*Subscale 3: positive disclosure attitudes and feelings* (4–20, n = 77)	14.13	4.03
**HIV Disclosure Intention**	Intention score (1–5, n = 90)	3.03	1.51
**HIV Stigma Scale**	*Negative self-image subscale* (3–12, n = 99)	6.11	2.40
**Social Support Questionnaire short form - SSQ6**	*Social support satisfaction subscale* (6–36, n = 76)	31.29	6.90
**Agentic and Communal Value Scale**	*Subscale: Agency* (12–108, n = 86)	70.74	17.31
	*Subscale: Communion* (12–108, n = 87)	79.54	16.26
**State Hope Scale**	Total hope score (6–48, n = 90)	35.00	7.42

### Reliability Analysis

Cronbach’s alpha for the total Adolescent HIV Disclosure Cognition and Affect scale was good (α = 0.78), with subscales demonstrating alphas of acceptable, very good and good levels (subscale 1, α = 0.70; subscale 2, α = 0.88; subscale 3, α = 0.78). Cronbach’s alpha was acceptable for the HIV Stigma Scale negative self-image subscale (α = 0.72). Cronbach’s alpha for the six-item Social Support Questionnaire Short form scale satisfaction subscale was excellent (α = 0.92). Cronbach’s alpha for both subscales of the Agentic and Communal Values scale was good (Agency, α = 0.82; Communion, α = 0.84). The State Hope scale had a good Cronbach’s alpha (α = 0.71).

### Criterion Validity

There was no significant difference on the CASE adherence between those who are virally suppressed (mean = 12.08, SD = 3.08, n = 76) and who are not virally suppressed (mean = 10, SD = 1.90, n = 6), *p* = 0.11, d = 0.81, indicating a large effect size.

There was no significant difference between disclosure affect and cognitions in those who had disclosed in the last 6 months (mean 59.40, sd 9.22, n = 15) versus those who had not (mean 58.29, sd 11.30, n = 52, p = 0.73, d = 0.11). There were significantly higher disclosure affect and cognitions scores in those who had disclosed to 4 or more people over their lifetime (mean 62.83, sd 9.58, n = 30) versus those who had disclosed to 0–3 people in their lifetime (mean 54.24, sd 10.41, n = 34, p = 0.001, d = 0.86). There was a significant positive relationship between disclosure affect and cognitions and disclosure intention (r [65] = 0.45, *p* < 0.001), a medium to large effect size. There was a significant negative relationship between disclosure affect and cognitions and HIV stigma (r [64] = -0.44, *p* < 0.001), a medium to large effect size.

There was a significant negative relationship between HIV stigma and wellbeing (*r* (85) = -0.53, 95% BCa: -0.67 – -0.35, *p* < 0.001), a large effect size. There was a significant positive relationship between social support and wellbeing (*r* [71] = 0.32, 95% BCa: 0.10–0.50, *p* < 0.01), a medium effect size. There was a significant negative relationship between social support and HIV stigma (r [72] = -0.31, 95% CI: -0.54 – -0.06, *p* = 0.01), a medium effect size. There was no relationship between social support and ART adherence (r [71] = -0.02 (95% BCa − 0.22–0.22, p = 0.84).

There was no significant difference in agency values in those who had not disclosed in the last 6 months (mean 70.39, sd 17.20, n = 75) versus those who had (mean 73.18, sd 18.66, n = 11, p = 0.62, d = 0.16). There was no significant difference in agency values in those who had disclosed to 4 or more people over their lifetime (mean 69.38, sd 16.28, n = 40) versus those who had disclosed to 0–3 people in their lifetime (mean 72.31, sd 18.96, n = 42, p = 0.46, d = 0.17). There was no significant relationship between agency values and disclosure intention (*r* (82) = 0.13, *p* = 0.25). There was no significant difference in communion values in those who had not disclosed in the last 6 months (mean 78.89, sd 15.78, n = 75) versus those who had (mean 83.58, sd 19.25, n = 12, p = 0.36, d = 0.27). There was no significant difference in communion values in those who had disclosed to 4 or more people in their lifetime (mean 78.85, sd 15.84, n = 41) versus those who had disclosed to 0–3 people in their lifetime (mean 80.33, sd 16.43, n = 43, p = 0.68, d = 0.09).There was no relationship between communion values and disclosure intention (*r* (83) = -0.09, 95% BCa: -0.31–0.15, *p* = 0.42).

There was a significant negative relationship between hope and HIV stigma (*r* (85) = -0.25, *p* = 0.02), a medium effect size, and a significant positive relationship between hope and wellbeing (r (85) = 0.36, 95% BCa: 0.16–0.53, *p* = 0.001), indicating a medium effect size.

## Discussion

The study addressed gaps in the literature concerning the lack of culturally and linguistically appropriate measures relevant to the lives of young adults living with HIV in Uganda. The translated and adapted measures assess ART adherence, HIV disclosure cognitions and affect, internalised HIV stigma, social support, personal values and hope. Overall, there was evidence that the adapted target measures were culturally and linguistically appropriate, and reliable, for young adults living with PAH in Uganda. One measure, the ACV, was, however, difficult to read in English, despite efforts to sensitively adapt this questionnaire.

The Adolescent HIV Disclosure Cognition and Affect Scale showed good levels of reliability. Subscale 2 and 3 showed comparably good levels of reliability between the Ugandan sample and a British sample of adolescents living with PAH [56], however subscale 1 (negative attitudes) was less reliable in the Ugandan sample. It is not clear why this might be, although the reliability was still acceptable. The agentic and communion subscales of the ACV scale [45] each showed good levels of reliability, and the internal consistency scores were higher than those in PAH British young adults aged 18–25 [47]. The SSQ-6 scale [57] showed excellent levels of reliability in PAH young people in Uganda, with a slight improvement over young adults living with HIV in the United States. The HIV stigma scale and State Hope scales both showed acceptable levels of reliability compared to samples of Swedish and US young adults respectively [73, 74].

Regarding criterion validity, relationships between the adapted measures and other key variables were found in many cases. HIV disclosure cognitions and affect were related to disclosure intention, consistent with a UK study with younger adolescents with PAH [28]. There was no relationship found between the AHDCAS measure and HIV disclosure in the last 6 months, however (despite finding an association between the total score and lifetime HIV disclosure). It may be that the measure of HIV disclosure behaviour in the last 6 months lacks validity, or that 6 months is too short a time period for such a low frequency event as HIV disclosure. A relationship was, however, found in the younger UK sample. Further exploration of the relationship between HIV disclosure and HIV disclosure cognitions and affect is warranted. A negative relationship between HIV disclosure cognitions and affect, and HIV stigma was found, whilst no such relationship was found in the UK study.

A positive relationship was found between social support satisfaction and well-being, whilst a negative relationship was seen with internal HIV stigma (perhaps reflecting a bidirectional causal relationship). This is consistent with previous work [41, 75, 76]. There was no relationship between social support satisfaction and self-reported ART adherence. Previous work has found an association between *family/caregiver* social support and ART adherence [40], whereas we used a *global* measure of social support *satisfaction.* In addition, the absence of a relationship may have been due to the generally high adherence scores, consistent with the high rates of viral suppression in the sample. There was no relationship between Agentic and Communion values and (a) disclosure intention, or (b) the number of people disclosed to in the last 6 months or over the lifetime. The intention finding contrasts with the work of Lehmann [47] who found that there was a relationship between agentic values and disclosure intention in a UK sample of young adults with PAH. It may be that there are different relationships between values and HIV disclosure in different contexts. It may also be that the true relationships between values, which are broad constructs, and specific relational behaviours such as HIV sharing, are weak. Finally, the complexity of the measure may have obscured true relationships between values and other variables.

HIV stigma was negatively related to psychological wellbeing and hope. It is important to note, however, that there may have been some measurement overlap between the HIV stigma and the wellbeing measure. Hope was positively associated with psychological wellbeing. Higher levels of hope were also associated with less stigma. There was, however, no relationship between CASE adherence and viral load, although the size of the effect suggests that this may have been due low statistical power.

There are some limitations of the study. First, the reliability and validity data reported are only preliminary given the limited sample size. Future work could investigate the predictive validity, convergent/divergent validity, factor structure and test-retest reliability of the measures in a larger sample. Secondly, the very high rates of viral suppression in the sample may not be representative of young adults living with PAH in Uganda or globally. There may also be differences between the sample and other young adults living with PAH on other characteristics (e.g., wellbeing, HIV stigma), which may limit external validity. However, it is important to note that in relation to HIV disclosure, the frequency of this event was low over the last 6 months and over the lifetime, as with other sample of young people living with HIV. One measure, the ACV, may need further clarification, although there was evidence of its reliability. Finally, it is not clear which language (English or Luganda) was used by the participants in completing the measures as they were given the option of either.

There are a number of strengths of the study. The study followed recommendations for cultural adaptation in the literature [54]. The use of an expert bilingual panel and cognitive interviewing with the target population was essential for clarifying confusing questions or response items on a conceptual or linguistic level. This cultural understanding was necessary to produce valid and reliable measures. The participants were sampled systematically (all young people meeting inclusion criteria at the study site were approached) with an acceptable response rate (61%).

In relation to research and practice implications, many of the adapted measures could be used in Uganda outside of the context of HIV. For example, the social support, hope, and personal values measures are not HIV-specific. In addition, it may be that the HIV-specific measures (CASE Adherence, AHDCAS, HIV stigma) can be used with other HIV populations in Uganda. Finally, the measures are brief enough to be used in clinical practice (e.g., the CASE adherence index) and in evaluations of psychosocial interventions (e.g., hope and HIV stigma).

### Electronic Supplementary Material

Below is the link to the electronic supplementary material.

Supplementary Material 1

## Data Availability

Available on request via authors.
