# Supplementary material for: Cultural Adaption, Translation, Preliminary Reliability and Validity of Key Psychological and Behavioural Measures for 18 to 25 Year-Olds Living with HIV in Uganda: A Multi-Stage Approach
Source: AIDS Behav. 2023 Oct 4;28(3):924–35. doi: 10.1007/s10461-023-04193-y (PMC10896775; doi:10.1007/s10461-023-04193-y)
Supplement: Supplementary file 1 — Supplementary Material 1 [file 10461_2023_4193_MOESM1_ESM.docx]

**Appendix A: The CASE Adherence index**

**Please ask each question and circle the corresponding number next to the answer.**

*Osabibwa okubuuza buli kibuuzo era olage ekyokudamu nga oteeka enkulungo kunamba eriranye ekyokudamu*

1. **How often do you feel that you have difficulty taking your HIV medications on time? By “on time” we mean no more than two hours before or two hours after the time your doctor told you to take it.**

*Buli luvanyuma lwabangaki lwowulira nga olina obukalubirivu mukumira edagalalyo ery’akawuka kamukenenya mubudde? “Mubudde” tutegeeza obutasuka sawa biri oba sawa biri eziyisewo kukiseera dokita kyeyakugamba okulimirako edagala*

1. **Never**/*Tekibangawo*
2. **Rarely**/*Kibaawo balirirwe*
3. **Most of the time**/*Emirun*di egisinga
   1. **All of the time**/*Ekiseera kyona*

1. **On average, how many days PER WEEK would you say that you missed at least one dose of your HIV medications?**

*Okugerageranya, naku meka buli wiki zoyinza okugamba nti wayosa omulundi nga gumu okumira edagala lyo ery’akawuka kamukenenya?*

- 1. **Everyday**/Buli lunaku
  2. **4-6 days/week** /*Naku 4-6 ewiki*
  3. **2-3 days/week/** *Naku 2-3 ewiki*
  4. **Once a week** /*Lunaku /teluwera ewiki*
  5. **Less than once a week** /*Olunaku teluwera*
  6. **Never** /*Tekibangawo*

1. **When was the last time you missed at least one dose of your HIV medications?**

*Di lwewasembayo okwosa okumira edagalalyo ery’akawuka kamukenenya omulundi nga gumu.*

- 1. **Within the past week**/ *Wiki ewedde*
  2. **1-2 weeks ago/** *Wiki 1-2 eziyise*
  3. **3-4 weeks ago/** *Wiki 3-4 eziyise*
  4. **Between 1 and 3 months ago**/ *Wakati mwezi 1 ne 3 egiyise*
  5. **More than 3 months ago**/ *Myezi gisuka 3 egiyise*
  6. **Neve**r/ *Tekibangawo*

**Appendix B: The Adolescent HIV Disclosure Cognition and Affect Scale**

| **How much do you agree with the following statements about telling people who do not know your status that you are HIV positive? (*Please tick once for each question)*** *Okiriziganya nakyo kyenkanawa kunyiriri zino wamanga okutegeeza abantu abatamanyi bikukwatako nti olina akawuka kamukeneya? (Osabibwa okugolola omulundi gumu kubuli kibuuzo)* | **Strongly disagree/** *Sikiriziganya nakyo dala* | **Disagree/** *sikiriziganya nakyo* | **Neither agree nor disagree/** *ndi wakati nzikiriziganya nakyo sikiriziganya nakyo;* | **Agree/** *Nzikiriziganya nakyo* | **Strongly agree/** *nzikiriziganya nakyo dala* |
| --- | --- | --- | --- | --- | --- |
| 1. **People will reject me or judge me**/*Abantu bajja kunegaana oba okunsalira omusango* |  |  |  |  |  |
| 1. **People will harm me**/ *Abantu bajja kuntuusako obulabe* |  |  |  |  |  |
| 1. **People will support me/** *Abantu bajja kumpagira.* |  |  |  |  |  |
| 1. **It will be easier to take my medication and** **look after my health**/ *Kijja kunyanguwira* *okumira edagala lyange nda birire nobulamu bwange* |  |  |  |  |  |
| 1. **I will feel better**./ *Nja kuwulira bulungi* |  |  |  |  |  |
| 1. **It is the right thing to do**/ *Kyekintu ekituufu okukola* |  |  |  |  |  |
| 1. **I’m worried they will tell others***/ Ndi mweralikirivu nti bajjakubulira abalala* |  |  |  |  |  |
| 1. **People will think negatively about my family**/ *Bantu bajja kulowoleza bubi abomumaka gange* |  |  |  |  |  |
| 1. **It will affect my relationship with them/** *Kijja kukosa enkolagana yange nabo* |  |  |  |  |  |
| 1. **People will worry about me/** *Abantu bajja kunelarikirira* |  |  |  |  |  |
| 1. **Most people in my situation would not tell other people that they’re HIV positive/** *Abantu abalala abali mumbera gyendimu tebajja kubulira bantu balala nti balina akawuka kamukenenya* |  |  |  |  |  |
| 1. **I am afraid to tell other people that I have HIV**/ *Ntya okubulira sabantu abalala nti nina akawuka kamukenenya* |  |  |  |  |  |
| 1. **I am confident that I can make the right choices about whom to share my HIV status/** *Ndi mwekakafu nti nsobola okukola okusalawo okutuufu kwani gwenina okubulirako kubinkwatako kukawuka kamukenenya* |  |  |  |  |  |
| 1. **I am confident that I can choose the right time and place to share my HIV status with others/** *Ndi mwekakafu nti nsobola okusalawo obudde obutuufu n’ekifo okutegeezako abalala ebinkwatako kukawuka kamukenenya* |  |  |  |  |  |
| 1. **I am confident that I can find the right words to say if I share my HIV status with others/** *Ndi mwekakafu nti nsobola okusalawo obudde obutuufu n’ekifo okutegeezako abalala ebinkwatako kukawuka kamukenenya* |  |  |  |  |  |
| 1. **I am confident that I can deal with how others respond if I share my HIV status with them/** *Ndi mwekakafu nti nsobula okwekolera kungeri abantu gebayinza okweyisamu bwemba mbategezezako bwenyimiridde kukawuka kamukenenya.* |  |  |  |  |  |
| 1. **I am confident that I can tell the other person what I feel and what I need if I tell them I am HIV positive*/*** *Ndi mwekakafu nti nsobola okutegeeza omuntu omulala bwempulira nakiki kyenetaaga singa mbabulira nti nina akawuka kamukenenya* |  |  |  |  |  |
| 1. **I am confident that I can get support from others if I tell them about my HIV status**/ *Ndi mwekakafu nti nsobola okufuna obuyambi okuva eri abantu abalala nsinga* *mbategeeza ebinkwatako kukawsuka kamukenenya* |  |  |  |  |  |
| 1. **I intend to tell someone new about my HIV status in the next 6 months/** *Nsubira okubulirayo omuntu omupya kubinkwatako kukawuka kamukenenya mubanga ery’emyezi mukaaga egijja* |  |  |  |  |  |

**Appendix C: The negative self-image subscale from the short form HIV Stigma Scale**

| **How much do you agree with the following statements?/***Okiririza kyenkanawa munyiriri zino wamanga?* | **Strongly disagree/** *Sikiriza dala* | **Disagree/** *sikikiriza* | **Agree/** *Nkiririza;* | **Strongly agree/** *Nkikiriza dala* |
| --- | --- | --- | --- | --- |
| 1. **I feel guilty because I have HIV/** *Mpulira nga gunsinze okuba nga nina akawuka kamukenenya* |  |  |  |  |
| 1. **I feel I’m not as good a person as others because I have HIV/** *Mpulira siri muntu mulungi nga abalala olwokuba nti nina akawuka kamukenenya.* |  |  |  |  |
| 1. **People’s attitudes about HIV make me feel worse about myself/** *Eneyisa zabantu eri akawuka kamukenenya zindetera okwekyawa* |  |  |  |  |

**Appendix D: The Social Support Questionnaire Short form – SSQ6**

**The following questions ask about people in your life who provide you with help or support. Each question has two parts. For the first part, list all the people you know, excluding yourself, who you can count on for help or support in the manner described. Write the person’s initials and their relation to you (see example). Do not list more than one person next to each of the numbers beneath the question.**

*Ebibuuzo ebidako bubuuzo kubantu mubulamubwo abakuwa obuyambi oba abakuyamba. Buli kibuuzo kirina ebitundu bibiri. Ekitundu ekisooka, kuwandiika bantu bona bomanyi, nga gwe wejjeko, boyinza okwesigamako okuyambibwa oba okukuwa obuyambi mungeri gyetunyonyodde. Wandiika enukuta ezisooka kulinya lw’omuntu nebwobayita (laba ekyokulabirako). Towandiika bantu basoba ku omu okulirana namba yemu eriko ekibuuzo*.

**For the second part, circle how satisfied you are with the overall support you have.**

**If you have no support for a question, circle the words “No one,” but still rate your level of satisfaction. Do not list more than nine people per question.**

**Please answer all the questions the best you can. All your responses will be kept confidential.**

*Mukitundu ekidako laga obumativubwo kubuyambi bwona bwofuna.*

*Singa oba nga tolina buyambi bwona bwofuna kukibuuzo ekikubuzidwa, laga ekyokudamu ekigamba nti “Tewali nomu,” naye osigale nga olaga obumativubwo. Towandiika bantu basoba mwenda mubuli kibuuzo*

*Gezako nga bwosobola okudamu ebibuuzo byona. Byona byonotugamba bijja kukumibwa nga byakyama.*

**Example:**

**Who do you know who you can trust with information that can get you into trouble?**

**No one 1.)T.N. (brother) 4.) D.N. (father) 7.)**

**2.) L.M. (friend) 5.) W.T. (employer) 8.)**

**3.) R.S. (friend) 6.) 9.)**

*Ekyokulabirako:*

*Ani gwomanyi gw’oyinza okwesiga n’obubaka obuyinza okukusuula mubuzibu?*

*Tewali muntu yenna*

*1.) T.N. (Mugandawange) 4.) D.N. (Taata) 7.)*

*2.) L.M. (Mukwano gwange) 5.) W.T. (Mukozesa) 8.)*

*3.) R.S. (Mukwano gwange) 6.) 9.)*

**How satisfied?**

**6 – very 5 - fairly 4 - a little 3 - a little 2 - fairly 1- very**

**satisfied satisfied satisfied dissatisfied dissatisfied dissatisfied**

*Obumativubwo bwenkanawa?*

6 *– Ndi mumativu nyo; 5 – Ndi mumativu; 4 – Ndi mumativu ekitonotono; 3 – Sili mumativu ekitonotono; 2 - Sili mumativu; 1- Sili mumativu dala*

1. **Who can you really count on to be dependable when you need help?**

*Ani gwoyinza okwesigamako okubeera omwesigwa nga wetaaga okuyambibwa?*

*Tewali nomu*

No one 1.) 4.) 7.)

2.) 5.) 8.)

3.) 6.) 9.)

**How satisfied?** *Obumativubwo bwenkanawa?*

**6 – Very/ 5 - fairly 4 - a little 3 - a little 2 - fairly 1- very**

**satisfied satisfied satisfied dissatisfied dissatisfied dissatisfied**

*6 – Ndi mumativu nyo 5 – Ndi mumativu; 4 – Ndi mumativu ekitonotono; 3 – Sili mumativu ekitonotono; 2 - Sili mumativu; 1- Sili mumativu dala*

1. **Who can you really count on to help you feel more relaxed when you are under pressure or tense?**

*Ani gwoyinza okwesigamako okukuyamba okukubudabuda okusobola okuwulira obulungi mukiseera nga olina ekinyigiriza omutimagwo?*

*Tewali nomu*

**No one 1.) 4.) 7.)**

**2.) 5.) 8.)**

**3.) 6.) 9.)**

**How satisfied?**

*Obumativubwo bwenkanawa?*

**6 – Very 5 - fairly 4 - a little 3 - a little 2 - fairly 1- very**

**satisfied satisfied satisfied dissatisfied dissatisfied dissatisfied**

*6 – Ndi mumativu nyo 5 – Ndi mumativu; 4 – Ndi mumativu ekitonotono; 3 – Sili mumativu ekitonotono; 2 - Sili mumativu; 1- Sili mumativu dala*

1. **Who accepts you totally, including both your worst and best qualities?**

*Ani akukiririza dala, mububi nemubulungi?*

*Tewali nomu*

**No one 1.) 4.) 7.)**

**2.) 5.) 8.)**

**3.) 6.) 9.)**

**How satisfied?**

*Obumativubwo bwenkanawa?*

**6 – Very 5 - fairly 4 - a little 3 - a little 2 - fairly 1- very**

**satisfied satisfied satisfied dissatisfied dissatisfied dissatisfied**

*6 – Ndi mumativu nyo 5 – Ndi mumativu; 4 – Ndi mumativu ekitonotono; 3 – Sili mumativu ekitonotono; 2 - Sili mumativu; 1- Sili mumativu dala*

**4. Who can you really count on to care about you, regardless of what is happening to you?**

*Ani gwoyinza okwesigamako okukulabirira, nga tafudde kukiki ekikutuuseko?*

*Tewali nomu*

**No one 1.) 4.) 7.)**

**2.) 5.) 8.)**

**3.) 6.) 9.)**

**How satisfied?**

*Obumativubwo bwenkanawa?*

**6 – Very 5 - fairly 4 - a little 3 - a little 2 - fairly 1- very**

**satisfied satisfied satisfied dissatisfied dissatisfied dissatisfied**

*6– Ndi mumativu nyo 5 – Ndi mumativu; 4 – Ndi mumativu ekitonotono; 3 – Sili mumativu ekitonotono; 2 - Sili mumativu; 1- Sili mumativu dala*

**5. Who can you really count on to help you feel better when you are feeling down-in-the-dumps?**

*Ani gwoyinza okwesigamako okukubudabuda owulire bulungi singa oba nga olimunyikavu?*

*Tewali nomu*

**No one 1.) 4.) 7.)**

**2.) 5.) 8.)**

**3.) 6.) 9.)**

**How satisfied?**

*Obumativubwo bwenkanawa?*

**6 – very 5 - fairly 4 - a little 3 - a little 2 - fairly 1- very**

**satisfied satisfied satisfied dissatisfied dissatisfied dissatisfied**

*6– Ndi mumativu nyo 5 – Ndi mumativu; 4 – Ndi mumativu ekitonotono; 3 – Sili mumativu ekitonotono; 2 - Sili mumativu; 1- Sili mumativu dala*

**6. Who can you count on to comfort you when you are very sad?**

*Ani gwoyinza okwesigamako okukubudabuda singa oba nga olina ekikujje mumbeera?*

*Tewali nomu*

**No one 1.) 4.) 7.)**

**2.) 5.) 8.)**

**3.) 6.) 9.)**

**How satisfied?**

*Obumativubwo bwenkanawa?*

**6 – very 5 - fairly 4 - a little 3 - a little 2 - fairly 1- very**

**satisfied satisfied satisfied dissatisfied dissatisfied dissatisfied**

*6– Ndi mumativu nyo 5 – Ndi mumativu; 4 – Ndi mumativu ekitonotono; 3 – Sili mumativu ekitonotono; 2 - Sili mumativu; 1- Sili mumativu dala*

**Appendix E: The Agentic and Communal Value Scale**

**Below are 24 different values that people rate of different importance in their lives. FIRST READ THROUGH THE LIST to familiarize yourself with all the values. While reading over the list, consider which ones tend to be most important to you and which tend to be least important to you. After familiarizing yourself with the list, rate the relative importance of each value to you as “A GUIDING PRINCIPLE IN MY LIFE.” It is important to spread your ratings out as best you can—be sure to use some numbers in the lower range, some in the middle range, and some in the higher range. Avoid using too many similar numbers. Work fairly quickly.**

*Bino wamanga byebintu 24 ebyenjawulo abantu byebagerageranyiza kumitendera egyenjawulo mubulamu bwabwe, SOOKA OSOME OLUKALALA okusobola okubitegeera obulungi. Bwoba osoma olukalala, laga ebyo byotwala okuba ebikulu gyoli nebyo byotwala okuba nga sibikulu gyoli. Bwomala okwekenenya olukalala, gerageranya obukulu bwabuli kintu nga “EKIMU KUBINTU OBULAMUBWO KWEBUTAMBULIRA.” Kyamugaso okulaga okugerageranyakwo nobwegendereza. Kozesa enamba ezisokerwako, eziri wakati, neziri wagulu. Wewale okukozesa enamba ezifanagana. Kino kikole mubwangu*

**Not Quite Highly**

**Important to important to me important to**

**me me me**

**1 - -- -- - 2 -- -- --- 3 -- ---- -- -- 4 --- ----- -- -- 5 -- -- ---- -- 6 -- -- -- -- --7--- -- --- -- 8 - - -------- - - 9**

*Sikyamugaso gyendi kyamugaso gyendi kyamugaso nyo gyendi*

*1----------2----------3----------4------------5----------6--------------7------------------8-------------9*

**——(01) WEALTH (financially successful, prosperous)**

*OBUGAGA (Okuyitamu mubyensimbi, okukulakulana)*

**——(02) PLEASURE (having one’s fill of life’s pleasures and enjoyments)**

*ESANYU (esanyu ly’omuntu nokunyumirwa)*

**——(03) FORGIVENESS (pardoning others’ faults, being merciful)**

*OKUSONYIWA (Okudiramu abantu olwebikyamu byebakoze, okubeera owekisa)*

**——(04) INFLUENCE (having impact, influencing people and events)**

*ETTUTUMU (okuba owomugaso, okukumakuma abantu)*

**——(05) TRUST (being true to one’s word, assuming good in others)**

*OBWESIGWA (okwesiga ekigambo ky’omuntu, okulowooleza abantu ebirungi)*

**——(06) COMPETENCE (displaying mastery, being capable, effective)**

*OBUSOBOZI (okulaga obusobozi, obusobozi, obulungi)*

**——(07) HUMILITY (appreciating others, being modest about oneself)**

*OBWETOWAZE (okusiima, nokwetowaliza abalala)*

**——(08) ACHIEVEMENT (reaching important goals)**

*OKUTUKIRIZA EBIRUBIRIRWA (okutukiriza ebirubirirwa byomugaso)*

**——(09) ALTRUISM (helping others in need)**

*OBUYAMBI (okuyamba abali mubwetavu)*

**——(10) AMBITION (high aspirations, seizing opportunities)**

*EKIRUBIRIRWA (Okulubirira okutukiriza, obubeera nemikisa egyokutukiriza)*

**——(11) LOYALTY (being faithful to friends, family, and group)**

*OBWESIGWA (okubeera omwesigwa eri abemikwano, abomumakago nabantu*

*abangi abawamu)*

**——(12) POLITENESS (courtesy, good manners)**

*OBUWOMBEEFU (okwewombeeka, empisa enungi)*

**——(13) POWER (control over others, dominance)**

*OBUYINZA (okukulembera abalala, okusinza obuyinza*

**——(14) HARMONY (good relations, balance, wholeness)**

*ENKOLAGANA ENUNGI (enkolagana enungi, okwesyisa obulungi)*

**——(15) EXCITEMENT (seeking adventure, risk, an exciting lifestyle)**

*OKUCAMUKA (Okunonya essanyu, obulabe, sobulamu obweyagaza)*

**——(16) HONESTY (being genuine, sincere)**

*OBWESIGWA (okubeera omwesigwa, obwesimbu)*

**——(17) COMPASSION (caring for others, displaying kindness)**

*OKUFAYO (Okulabirira abalala, ssokulaga ekisa)*

**——(18) STATUS (high rank, wide respect)**

*EDAALA (edaala lyawagulu, ekitibwsa kyamanyi)*

**——(19) KINDNESS (being considerate and respectful toward others)**

*Ekisa (Obuntu bulamu, ekitiibwa, okuba ne kisa)*

**——(20) AUTONOMY (independent, free of others’control)**

*OKWEMALIRIRA (okwefuga, okwetwala)*

**——(21) EQUALITY (human rights and equal opportunity for all)**

*OMWENKANONKANO (eddembe lyobuntu nemikisa egyenkanankana eri buli omu)*

**——(22) RECOGNITION (becoming notable, famous, or admired)**

*OKUMANYIKA (Okumanyika, obumanyifu oba okwegombesa)*

**——(23) TRADITION (showing respect for family and cultural values)**

*OBUWANGWA (okulaga abenganda ekitibwa nobuwangwa)*

**——(24) SUPERIORITY (defeating the competition, standing on top)**

*OKUBEERA KUNTIKO (okuwangula empaka, okubeera kuntiko)*

**Appendix F: The State Hope Scale**

**Read each item carefully. Using the scale shown below, please select the number that best describes *how you think about yourself right now* and put that number in the blank before each sentence. Please take a few moments to focus on yourself and what is going on in *your life at this moment.* Once you have this “here and now” set, go ahead and answer each item according to the following scale:**

*Soma buli kimu nobwegendereza. Nga okozesa ekipimo ekyo wamanga, osabibwa okulonda enamba enyonyola nga bwewelowoozako mukiseera kino era oteke enamba eyo kulunyiriri kwegendera. Osabibwa okutwala obudde nga wetunulira nebigenda mumaaso mubulamubwo kukiseera kino. Bwomala okukola kino “wano kukiseera kino”, genda mumaaso nokudamu buli kintu okusinziira kubipimo bino wamanga:*

| **1** | **2** | **3** | **4** | **5** | **6** | **7** | **8** |
| --- | --- | --- | --- | --- | --- | --- | --- |
| **Definitely False/** *Sikituufu nakamu* | **Mostly False/** *sikituufu dala* | **Somewhat False/** *Sikituufu;* | **Slightly False/** *Sikituufu ekitonotono* | **Slightly True/** *kituufu ekitonotono* | **Somewhat True/** *kituufu* | **Mostly True/** *kituufu dala,* | **Definitely True/** *kituufu dala dala.* |

_____ **1. If I should find myself in a jam, I could think of many ways to get out of it**

*Singa nesanga mukiseera ekizibu, ndowooza kungeri nyingi ezokukivunuka*

_____ **2**. **At the present time, I am pursuing my goals with energy**

*Mukiseera kino, Nkola namanyi okutukiriza ebirubirirwa byange*

_____ **3**. **There are lots of ways around any problem that I am facing now**

*Waliwo engeri nyingi kubuli kiizibu kyendimu kati*

_____ **4. Right now, I see myself as being pretty successful**

*Mukiseera kino, ndaba nga emikisa gyange mitangaavu.*

_____ **5. I can think of many ways to reach my current goals**

*Nsobola okulowooza kungeri nyingi okutuuka kubirubirirwa bwange byenina kati*

_____ **6. At this time, I am meeting the goals that I have set for myself**

*Kukiseera kino, ntukiriza birubirirwa byeneteredewo nze kenyini*
